# Thermochemical Properties of Hydrophilic Polymers from Cashew and Khaya Exudates and Their Implications on Drug Delivery

**DOI:** 10.1155/2016/7496585

**Published:** 2016-11-20

**Authors:** Emmanuel O. Olorunsola, Partap G. Bhatia, Babajide A. Tytler, Michael U. Adikwu

**Affiliations:** ^1^Department of Pharmaceutics and Pharmaceutical Technology, University of Uyo, Uyo, Nigeria; ^2^Department of Pharmaceutics and Pharmaceutical Microbiology, Ahmadu Bello University, Zaria, Nigeria; ^3^Department of Pharmaceutics and Pharmaceutical Microbiology, Usmanu Danfodiyo University, Sokoto, Nigeria; ^4^University of Abuja, Abuja, Nigeria

## Abstract

Characterization of a polymer is essential for determining its suitability for a particular purpose. Thermochemical properties of cashew gum (CSG) extracted from exudates of* Anacardium occidentale L.* and khaya gum (KYG) extracted from exudates of* Khaya senegalensis* were determined and compared with those of acacia gum BP (ACG). The polymers were subjected to different thermal and chemical analyses. Exudates of CSG contained higher amount of hydrophilic polymer. The pH of 2% w/v gum dispersions was in the order KYG < CSG < ACG. Calcium was the predominant ion in CSG while potassium was predominant in KYG. The FTIR spectra of CSG and KYG were similar and slightly different from that of ACG. Acacia and khaya gums exhibited the same thermal behaviour which is different from that of CSG. X-ray diffraction revealed that the three gums are the same type of polymer, the major difference being the concentration of metal ions. This work suggests the application of cashew gum for formulation of basic and oxidizable drugs while using khaya gum for acidic drugs.

## 1. Introduction

Plant gums are produced either as exudates, seed gums, seaweed gums, or pectin [1]. Many tropical plants produce gum as exudates, and these include* Acacia senegal, Anacardium occidentale* L., and* Khaya senegalensis* [1, 2]. Gummy exudates are produced by a process called gummosis which is the secretion of gluey substances from plant stem and trunk. The sticky substance, on exposure to air, solidify to form amorphous translucent solid called gum [3].

Cashew,* Anacardium occidentale* L., belongs to the family Anacardiaceae [2]. It is native to Brazil and grows in many tropical and subtropical countries including Nigeria. It can grow on soils that are considered too poor or too stony for other crops to grow. However, it prefers a well drained sandy-loamy soil. The tree tolerates a wide range of soil acidity [4]. Cashew gum is light to dark brown in colour. It has a pH range of 3.80 to 5.22 depending on the source and duration of storage. The gum contains 9.8–13.2% moisture, 1.9–4.8% insoluble matter, 0.5–1.2% total ash, 1.27–1.80% protein, 9.6–21.0% sugar, and 0.21–2.26% phenol [5]. In terms of sugar composition, it is composed of 61% galactose, 14% arabinose, 7% rhamnose, 8% glucose, 5% glucuronic acid, and <2% other sugar residues. The gum has a highly branched galactan framework made up of chains of 1,3-linked *β*-D-galactopyranosyl units interspersed with *β*- 1, 6-linked bonds [6].

*Khaya senegalensis* (commonly called African mahogany) belongs to the Family Meliaceae. The plant is widely distributed in the rain forest and scattered within the higher rainfall savannah regions. It is characterized and recognized by its evergreen crown [7]. Khaya gum occurs in long thin glass-like translucent fragments. It is colourless to light brown and its mucilage has an acidic pH of about 4.2. It is insoluble in ethanol and acetone and only slightly soluble in water [7]. It is a hydrophilic polymer with a galactan core in which the 1,3-linked *β*-D-galactopyranosyl residues are concentrated in the inner chains. According to Edmund [8], the gum shares a lot of similarities with acacia gum.

Cashew gum has been evaluated and subsequently suggested for use as a binder in tablet formulations [9], as an agglutinant for capsules and pills in place of gum Arabic [6], as a gelling agent in topical gel formulation [10], and as a suspending and emulsifying agent [11]. Similarly, khaya gum has been evaluated and subsequently suggested for use as a binder in tablet formulation [12] and as a suspending agent in liquid formulation [13]. Therefore, cashew gum and khaya gum have been recommended in several instances for use as alternatives to standard acacia gum.

Cashew gum, khaya gum, and acacia gum are related in that they are all hydrophilic polymers with galactan core and are all obtained from plant exudates [6–8]. Acacia gum is well characterized and it is the official standard. In contrast, most of the information obtained from literature on work carried out till date on characterization of the other two polymers concentrated on the physical properties and carbohydrate monomer composition of the polymers. This work was aimed at evaluating the thermal and chemical properties of cashew and khaya gums and comparing them with those of standard acacia gum. It provides information about the suitability of these hydrophilic polymers as pharmaceutical excipients.

## 2. Materials and Methods

### 2.1. Materials

Crude cashew exudates were collected from* Anacardium occidentale* L. trees in Abuja, Nigeria, authenticated by the taxonomist in the Department of Biological Sciences, University of Abuja, Abuja, Nigeria, and issued with voucher number UNIABUJA 150. Crude khaya exudates were collected from* Khaya senegalensis* trees in Abuja, Nigeria, authenticated by the same taxonomist and issued with voucher number UNIABUJA 152. Standard* Acacia senegal* gum (BDH Chemicals, Poole, England) was used as obtained. Ethanol (BDH Chemicals, Poole, England), acetone (Merck, Germany), and diethyl ether (Sigma-Aldrich, Germany) were used as obtained.

### 2.2. Extraction and Purification of the Polymers

The cashew tree exudates were cleaned by removing the particles of the bark and other extraneous materials by hand and then purified using the modified form of the method described by Ofori-Kwakye et al. [2]. A 500 g quantity of the exudates was added to 1 L of distilled water. The mixture was homogenized using a laboratory blender and then left for 24 h to ensure complete dissolution. The mucilage was screened with muslin cloth to obtain particulate-free slurry. The gum was precipitated using absolute ethanol and defatted using diethyl ether. The precipitated gum was air-dried for 3 h then at 50°C in a hot air oven (Gallenkamp, Germany) for 24 h. It was pulverized using laboratory blender (Model 38BL40, Christison, United Kingdom).

The khaya tree exudates were cleaned by removing extraneous materials by hand. A 500 g quantity of the exudates was purified using the method described for cashew gum. However, 2 L of distilled water was used and time period of 48 h was allowed for gum dissolution. The mucilage was screened with muslin cloth and the gum was precipitated using twice the volume of acetone and defatted using 500 mL of diethyl ether. The gum was dried and then pulverized.

### 2.3. Determination of Percentage Yield of the Purified Gums

The dried, precipitated, and purified gums obtained from the exudates were weighed and the percentage yields were expressed relative to the initial weight of the crude exudates.

### 2.4. Determination of Moisture Content

The percentage of moisture content of 2 g sample of each polymer was analysed using electronic moisture analyser (Type MB 35, OHAUS, Switzerland).

### 2.5. Determination of pH

A 2 g sample of each polymer was dispersed in sufficient deionized water and the volume was made to 100 mL with more of the deionized water to produce a 2% w/v dispersion of the polymer. The pH of the dispersion was determined 24 h after preparation using a pH meter.

### 2.6. Atomic Absorption Spectrophotometry (AAS)

The AAS was carried out using the method described by Ofori-Kwakye et al. [2]. One mL of the digest was used to determine the amount of lead, magnesium, copper, and calcium at wavelengths of 283.30 nm, 285.21 nm, 324.75 nm, and 422.67 nm, respectively, using an Atomic Absorption Spectrophotometer (Perkin Elmer Analyst 400, Perkin Elmer, USA) fitted with an acetylene flame. Also, 2 mL of the digest was used for the determination of sodium and potassium using a flame photometer (Jenway Model PFP7, United Kingdom) operated on methane gas.

### 2.7. Differential Scanning Calorimetry (DSC)

DSC analysis was carried out on 3 mg sample in a 40 *μ*L Al crucible using a DSC–204FI machine (NETZSCH Co., Germany). The scanning was done at 20°C/min heating rate over a temperature range of 0–500°C under nitrogen environment.

### 2.8. Fourier Transform Infrared (FTIR) Spectrophotometry

Samples of the polymers were prepared in KBr disks in a hydrostatic press at 6–8-ton pressure. FTIR spectra of these prepared samples were recorded at scanning range of 350 to 5000 cm^−1^ using a spectrophotometer (model 8400S, Shimadzu Corporation, Koyto, Japan).

### 2.9. X-Ray Diffraction (XRD)

Powder X-ray diffraction pattern of the samples was recorded on an X-ray diffractometer (PANalytical Spectris Pvt. Ltd., Singapore) using a copper target at voltage of 40 KV and a current of 30 mA over scanning range of 10 to 120°2 Theta.

### 2.10. Data Analysis

Data obtained were expressed as mean ± standard error of the mean. Statistical analysis was done using one-way analysis of variance followed by Turkey-Kramer multiple comparison test using GraphPad Instat-3 software. Significance of difference was taken at *p values* < 0.05.

## 3. Results

### 3.1. Some Physicochemical Properties

Some physicochemical properties of the three hydrophilic polymers are shown in Table 1. The cashew plant exudates gave a higher yield of gum compared to the khaya plant exudates. There was no significant difference in the moisture content of the three polymers. The pH of the 2% w/v dispersion of the gums was in the order KYG < CSG < ACG and the differences were significant.

### 3.2. Metal Ion Content

The metal ion contents of the different polymers are illustrated in Table 2. Cashew gum had the highest concentration of sodium ion while KYG had the lowest concentration. Khaya gum had the highest potassium ion concentration while ACG had the lowest value. Potassium was observed to be the predominant monovalent ion in khaya gum while sodium was observed to be the predominant in acacia gum. Cashew gum had the highest magnesium ion concentration while khaya gum had the lowest value. Calcium ion was observed to be the predominant ion in CSG. There was a significant difference in the concentration of calcium ion in the different polymers. Khaya gum had a significantly higher concentration of copper ion compared to cashew gum. All the polymers were observed to be free of lead ion.

### 3.3. Differential Scanning Thermograms

**Figure 1:**
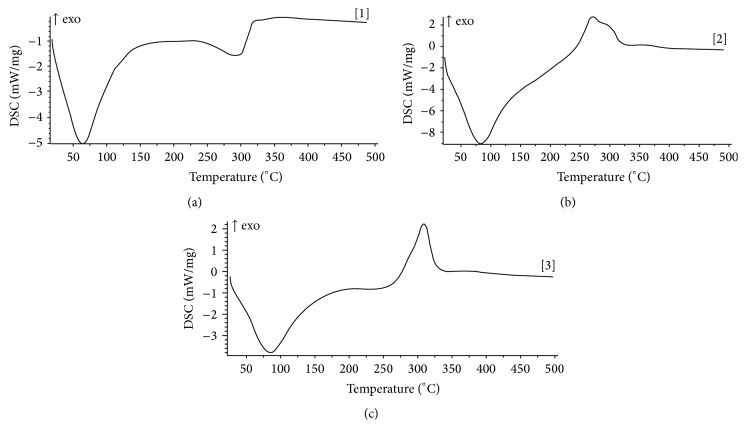
DSC thermograms of (a) cashew gum, (b) khaya gum, and (c) acacia gum.

The DSC thermograms of the polymers are shown in Figure 1. The thermogram of CSG showed a sharp endothermic transition between 20 and 100°C with a peak at 70°C followed by a diffuse endotherm which peaked at 305°C. There was an endothermic transition having peak at 90°C followed by a diffuse exotherm which peaked at 275°C in the thermogram of KYG. In the thermogram of ACG, an endotherm which peaked at 85°C was observed between 20 and 200°C. This was immediately followed by a sharp exotherm between 275 and 335°C. The enthalpy changes in the thermograms of the three polymers are shown in Table 3.

### 3.4. Fourier Transform Infrared (FTIR) Spectra

**Figure 2:**
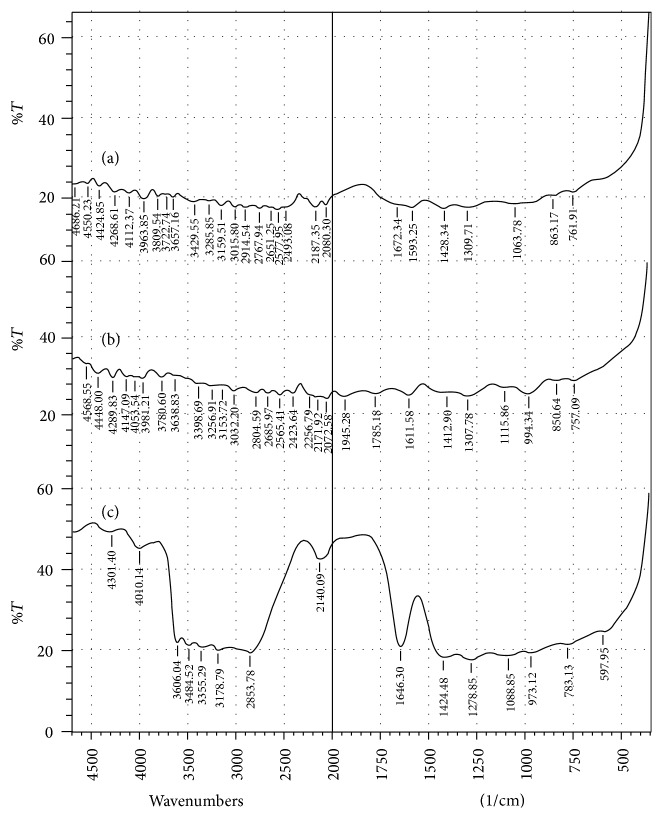
FTIR spectra of (a) cashew gum, (b) khaya gum, and (c) acacia gum.

The FTIR spectra of the different polymers are shown in Figure 2. The spectra of cashew gum and khaya gum were similar, the peak of highest intensity being 4,550.23 cm^−1^ for cashew gum and 4,568.55 cm^−1^ for khaya gum. The first absorption peak was 761.91 cm^−1^ for CSG while it was 757.09 cm^−1^ for KYG. The absorption pattern for CSG and KYG was different from that of ACG. The first peak for ACG was noticed at 597.95 cm^−1^ and the peak at 4,301.4 cm^−1^ had the highest intensity.

### 3.5. X-Ray Diffraction (XRD) Spectra

**Figure 3:**
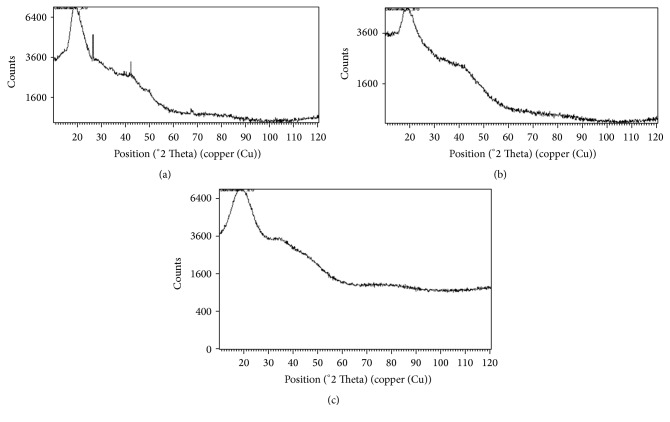
XRD spectra of (a) cashew gum, (b) khaya gum, and (c) acacia gum.

The XRD spectra of the different polymers are shown in Figure 3. The three polymers had major peak of height > 6400 counts lying between 20 and 22 °2 Theta. Cashew gum had two other peaks at 26.50 and 42.34 °2 Theta corresponding to calcium pantoate. The major peak of KG spectrum is shorter and more flattened.

## 4. Discussion

The exudates of* Anacardium occidentale* L. contains higher amount of gum compared to that of* Khaya senegalensis* (Table 1). The yield of 48.0% of cashew gum is lower than the value reported (78.5%) by Ofori-Kwakye et al. [2]. It was claimed that the high yield was due to the nature of the precipitating solvent (ethanol) used. Ethanol was used in this present work, yet the yield is not higher than that obtained by Kumar et al. [10] who used acetone as precipitating solvent. Therefore, the difference in yield could not be due to solvent alone but also due to other factors such as the source of the exudates.

The moisture content was in the order ACG < CSG < KYG, though the difference was not significant. Water is known to catalyze many degradative processes including hydrolysis and microbial attack. The higher the moisture content, the more liable the polymer to degradation [14]. Therefore, the difference in their stability in the dry state might not be significant.

The three polymers have pH in the acidic range showing their commercial and pharmaceutical importance. Basic excipients promote oxidation of susceptible drugs when used for their formulations [15]. Therefore, acidic and neutral hydrocolloids are more widely used for pharmaceutical formulations [7]. Interactions between acidic drugs and basic excipients and vice versa have also been reported by many researchers [16]. Hence, CSG might possess better drug/excipient compatibility as the pH is closer to neutrality.

Calcium ion being the predominant ion in cashew gum is in agreement with the work of Ofori-Kwakye et al. [2]. The concentration of potassium and calcium ions might be a good distinguishing factor between khaya and cashew gums. While potassium was the predominant ion (1600 mg/kg) in khaya gum, calcium was the most predominant (2397 mg/kg) in cashew gum (Table 2). Interactions between drugs and pharmaceutical excipients based on their ionic composition were also reported by Zhou [16]. According to him, indomethacin is incompatible with sodium bicarbonate and ibuprofen is incompatible with magnesium oxide while norfloxacin is incompatible with magnesium stearate. Cashew gum having significantly higher concentration of sodium and magnesium ions might interact with these drugs.

Copper (II) ion is a strong oxidizing agent and catalyzes oxidation of susceptible substances [15]. Khaya gum has a significantly higher concentration of copper (II) ion and might be more vulnerable to autooxidation. It is also less suitable for formulation of drugs that are susceptible to oxidation. Lead is a highly toxic metal and it is absent in all the polymers. This factor and the low concentration of copper ion in all the polymers support the statement that gums are safe pharmaceutical excipients [3].

The first endotherm of the thermogram of CSG (illustrated in Figure 1) can be ascribed to the enthalpy relaxation of the polymer [17]. No exothermic transition was observed. Therefore, there was no clear degradation of the polymer by heating up to 500°C. The second endotherm can be ascribed to melting of the polymer [18] and the peak of the endotherm (305°C) represents its melting point. The latent heat of melting of the polymer was 286.36 J/g (Table 3). This is the amount of energy required to melt a unit gram of the polymer [19]. The observed polymer melting instead of polysaccharide degradation might be due to high inorganic composition of the polymer as revealed by the atomic absorption spectrophotometry. Melting transition is characteristic of crystalline domain [20].

The endotherm of the thermogram of KYG (also illustrated in Figure 1) can be ascribed to the enthalpy relaxation of the polymer [17]. Therefore, it could be said that the polymer experienced enthalpy relaxation between 20 and 250°C. The diffuse exotherm represents polysaccharide degradation [21] and the observed enthalpy change of −454.50 J/g shows that 454.50 J of heat was lost as 1 g of the polymer degraded.

The enthalpy relaxation of acacia gum was illustrated by the endothermic transition between 20 and 200°C [17]. Just like in khaya gum, the exotherm can be ascribed to polysaccharide degradation [21]. The enthalpy change of −363.63 J/g shows that 363.63 of heat was lost as 1 g of the polymer degraded. Relating this to the thermogram of khaya gum, it shows that a smaller amount of heat was lost as acacia gum degraded.

Enthalpy relaxation is an endothermic process as it involves heat flow to the system. It is associated with increase in temperature and hence involves change in heat capacity. It is, therefore, a second-order reaction. In some instances, when the right temperature is reached after relaxation, particles attain an orderly arrangement forming crystals in the process referred to as crystallization. Crystallization involves heat loss. Hence, it is an exothermic transition [18]. Since it takes place at a constant temperature, it is a first-order reaction [19]. As heating continues beyond crystallization, a temperature is reached when the crystals move out of the orderly arrangement in a process called melting. Unlike most polysaccharides, cashew gum experienced endothermic transition of melting instead exothermic transition of degradation. Just like crystallization, melting takes place at a constant temperature. It is thus a first-order transition [18].

The enthalpy relaxation of both KYG and ACG that were accompanied by exothermic transition of degradation reflects their polymeric nature [21]. It can be concluded that when these polymers were heated, they were converted from the amorphous to pseudoamorphous form in the process of enthalpy relaxation. Further heating resulted in polysaccharide breakdown reflected by the exothermic transition.

The Fourier transform infrared spectra of cashew gum and khaya gum are similar (Figure 2). The first absorption peak of 761.91 cm^−1^ in cashew gum and 757.09 cm^−1^ in khaya gum correspond to the presence of aromatic nuclei in the two polymers [22]. The two spectra have similar peak pattern and may contain similar functional groups.

The prominent peak of cashew gum at 761.91 cm^−1^ could be assigned to C-H bending vibration of substituted aromatic hydrocarbon while those at 2914.54–3265.65 cm^−1^ could be attributed to stretching vibration of the aromatic C-H. The peaks at 1063.78 and 1309.71 cm^−1^ could be assigned to O-H bending and C-O stretching of ether and alcohol. The peaks at 2577.95 and 2651.25 cm^−1^ could be assigned to stretching vibration of intramolecularly bonded O-H group, while 3657.16 cm^−1^ peak could be assigned to stretching vibration of free O-H based on infrared spectrophotometry bands of selected functional groups given by Coutts [22].

In the FTIR spectrum of khaya gum, the peak at 757.09 cm^−1^ could be assigned to aromatic C-H bending vibration while those at 3032.20–3256.91 cm^−1^ could be attributed to stretching vibration of C-H in the substituted aromatic hydrocarbon. The peaks at 1115.86 and 1307.78 cm^−1^ could be assigned to O-H bending and C-O stretching of ether and alcohol. The peaks at 2561.41 and 2685.97 cm^−1^ could be assigned to stretching vibration of bonded hydroxyl group while 3638.83 cm^−1^ could be assigned to stretching vibration of free O-H [22].

The absorption at 597.95 cm^−1^ (<600 cm^−1^) found in acacia gum was not found in cashew nor khaya gum. However, peaks below 600 cm^−1^ are not used in characterization. In the FTIR spectrum of acacia gum, the peak at 783.13 cm^−1^ could be assigned to aromatic C-H bending vibration while those at 2853.78 and 3178.79 cm^−1^ could be assigned to C-H stretching vibration of the aromatic hydrocarbon. The peaks at 1088.85 and 1278.85 cm^−1^ could be assigned to O-H bending and C-O stretching of ether and alcohol. The peak at 3484.52 cm^−1^ could be assigned to the stretching of bonded O-H group while 3606.04 cm^−1^ could be assigned to stretching vibration of free O-H group (Figure 2) based on specifications of Coutts [22].

From the X-ray diffraction patterns (Figure 3), the major peaks of CSG, KYG, and ACG lying at the same point are an indication that the three gums are the same type of polymer. The abundance of calcium in cashew gum was confirmed by the two additional peaks at 26.49 and 42.34 °2 Theta which correspond to the presence of calcium pantoate. The shorter and more flattened peak of KYG indicates the presence of impurities [23]. Organic molecules are large and their crystals have large unit cells. Their interplanar spacings are, therefore, large. The large spacing gives rise to diffraction peaks at small values of °2 Theta. This is particularly true for amorphous materials [24]. All the polymers have single major peak below 25 °2 Theta which is on the low side. They are, therefore, amorphous organic materials.

## 5. Conclusion

Exudate of cashew plant contains higher amount of gum compared to that of khaya plant. The polymers are good and safe pharmaceutical excipients with pH in the acidic range. They are free of lead ion and are distinguishable by the level of different metal ions present. Khaya gum and acacia gum have similar thermal behaviour which is different from that of cashew gum. The three gums are the same type of polymer, the only significant difference being the concentration of the different metal ions. This work suggests the application of cashew gum for formulation of basic drugs while using khaya gum for acidic drugs. Cashew gum might also be better as excipient for formulation of drugs that are susceptible to oxidation. The suitability of each polymer can be verified by utilizing and investigating them for delivery of the different drugs.

## Figures and Tables

**Table 1:** Some physicochemical properties of the polymers.

Parameter	CSG	KYG	ACG
Yield (%)	48.0	25.0	ND
Moisture content (%)	6.59 ± 0.26	6.62 ± 0.41	6.52 ± 1.38
pH	4.04 ± 0.01	3.38 ± 0.00	4.61 ± 0.01

ND: not determined, CSG: cashew gum, KYG: khaya gum, and ACG: acacia gum.

**Table 2:** Metal ion content of the polymers.

Ion	Concentration (mg/kg)		
	CSG	KYG	ACG
Na^+^	1,500 ± 0.20	300 ± 0.11	900 ± 0.32
K^+^	550 ± 0.25	1,600 ± 0.50	140 ± 0.17
Mg^2+^	1,375 ± 0.19	2.85 ± 0.15	441 ± 0.15
Ca^2+^	2,397 ± 0.26	18 ± 0.22	705 ± 0.15
Cu^2+^	3.1 ± 0.04	9.0 ± 0.09	0.00 ± 0.00
Pb^2+^	0.00 ± 0.00	0.00 ± 0.00	0.00 ± 0.00

CSG: cashew gum, KYG: khaya gum, and ACG: acacia gum.

**Table 3:** Enthalpy changes in the thermograms of the polymers.

Polymer	Enthalpy of first transition	Enthalpy of second transition
CSG	909.09 J/g (endotherm)	286.36 J/g (endotherm)
KYG	2833.30 J/g (endotherm)	−454.50 J/g (exotherm)
ACG	1060.61 J/g (endotherm)	−363.63 J/g (exotherm)

CSG: cashew gum, KYG: khaya gum, and ACG: acacia gum.
